# Bis[4-(dimethyl­amino)pyridinium] tetra­bromidobis(4-chloro­phen­yl)stannate(IV)–4-bromo­chloro­benzene (1/1)

**DOI:** 10.1107/S1600536809030232

**Published:** 2009-08-08

**Authors:** See Mun Lee, Kong Mun Lo, Hapipah Mohd Ali, Ward T. Robinson

**Affiliations:** a Department of Chemistry, University of Malaya, 50603 Kuala Lumpur, Malaysia

## Abstract

In the title compound, (C_7_H_11_N_2_)_2_[SnBr_4_(C_6_H_4_Cl)_2_]·C_6_H_4_BrCl, the Sn^IV^ atom in the tetra­bromidobis(4-chloro­phen­yl)stannate(IV) anion lies on a centre of inversion. The distances between the 4-(dimethyl­amino)pyridinium N atom and the Br atoms of the anion are 3.450 (2) and 3.452 (2) Å, suggesting weak hydrogen bonding. The 4-bromo­chloro­benzene solvent mol­ecule, which is a bromination by-product from the reaction, is disordered about a twofold rotation axis with approximately equal occupancy.

## Related literature

For related structures, see Lo & Ng (2009 ▶); Koon *et al.* (2009 ▶); Yap *et al.* (2008 ▶).
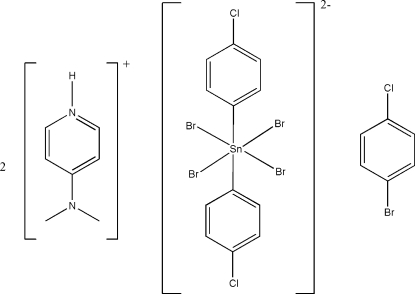


## Experimental

### 

#### Crystal data

(C_7_H_11_N_2_)_2_[SnBr_4_(C_6_H_4_Cl)_2_]·C_6_H_4_BrCl
*M* *_r_* = 1099.22
Triclinic, 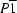

*a* = 8.7692 (18) Å
*b* = 10.128 (2) Å
*c* = 11.407 (2) Å
α = 111.16 (3)°
β = 93.38 (3)°
γ = 92.85 (3)°
*V* = 940.4 (3) Å^3^
*Z* = 1
Mo *K*α radiation
μ = 6.23 mm^−1^
*T* = 100 K
0.45 × 0.26 × 0.19 mm

#### Data collection

Bruker APEXII CCD area-detector diffractometer
Absorption correction: multi-scan (*SADABS*; Sheldrick, 1996 ▶) *T*_min_ = 0.169, *T*_max_ = 0.384 (expected range = 0.135–0.306)
7255 measured reflections
4265 independent reflections
3919 reflections with *I* > 2σ(*I*)
*R*_int_ = 0.019

#### Refinement

*R*[*F* ^2^ > 2σ(*F* ^2^)] = 0.023
*wR*(*F* ^2^) = 0.062
*S* = 1.05
4265 reflections
207 parameters
H-atom parameters constrained
Δρ_max_ = 0.77 e Å^−3^
Δρ_min_ = −1.12 e Å^−3^

### 

Data collection: *APEX2* (Bruker, 2008 ▶); cell refinement: *SAINT* (Bruker, 2008 ▶); data reduction: *SAINT*; program(s) used to solve structure: *SHELXS97* (Sheldrick, 2008 ▶); program(s) used to refine structure: *SHELXL97* (Sheldrick, 2008 ▶); molecular graphics: *SHELXTL* (Sheldrick, 2008 ▶); software used to prepare material for publication: *publCIF* (Westrip, 2009 ▶).

## Supplementary Material

Crystal structure: contains datablocks I, global. DOI: 10.1107/S1600536809030232/hg2523sup1.cif

Structure factors: contains datablocks I. DOI: 10.1107/S1600536809030232/hg2523Isup2.hkl

Additional supplementary materials:  crystallographic information; 3D view; checkCIF report

## Figures and Tables

**Fig. 1.**
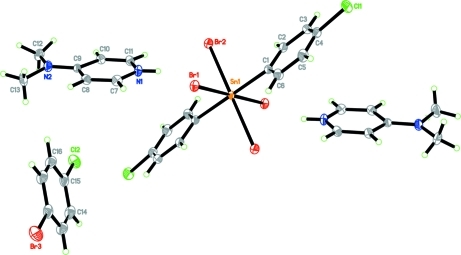
The molecular structure of bis[4-(dimethylamino)pyridinium] tetrabromidobis(4-chlorophenyl)stannate(IV) 4-bromochlorobenzene, showing 50% probability displacement ellipsoids and the atom numbering. Hydrogen atoms are drawn as spheres of arbitrary radius.

## supplementary crystallographic information

### Experimental

Tetra(4-chlorophenyl)tin (0.57 g, 1 mmol) and 4-dimethylaminopyridine hydrobromide perbromide (0.40 g, 1 mmol) was dissolved in absolute ethanol (25 ml) and refluxed for six hours. The solution was filtered and colourless crystals were isolated upon cooling.

### Refinement

Hydrogen atoms were placed at calculated positions (C–H 0.95 to 0.98 Å) and were treated as riding on their parent carbon atoms, with *U*(H) set to 1.2–1.5 times *U*(C,*N*). N—H was refined and placed in the calculated position of N—H 0.88 ± 0.01 Å.

### Crystal data

**Table:**

(C_7_H_11_N_2_)_2_[SnBr_4_(C_6_H_4_Cl)_2_]·C_6_H_4_BrCl	*Z* = 1
*M**_r_* = 1099.22	*F*(000) = 530
Triclinic, *P*1	*D*_x_ = 1.941 Mg m^−^^3^
Hall symbol: -P 1	Mo *K*α radiation, λ = 0.71073 Å
*a* = 8.7692 (18) Å	Cell parameters from 6036 reflections
*b* = 10.128 (2) Å	θ = 2.2–30.5°
*c* = 11.407 (2) Å	µ = 6.23 mm^−^^1^
α = 111.16 (3)°	*T* = 100 K
β = 93.38 (3)°	Block, colourless
γ = 92.85 (3)°	0.45 × 0.26 × 0.19 mm
*V* = 940.4 (3) Å^3^	

### Data collection

**Table:**

Bruker APEXII CCD area-detector diffractometer	4265 independent reflections
Radiation source: fine-focus sealed tube	3919 reflections with *I* > 2σ(*I*)
graphite	*R*_int_ = 0.019
ω scans	θ_max_ = 27.5°, θ_min_ = 1.9°
Absorption correction: multi-scan (*SADABS*; Sheldrick, 1996)	*h* = −11→8
*T*_min_ = 0.169, *T*_max_ = 0.384	*k* = −13→13
7255 measured reflections	*l* = −14→13

### Refinement

**Table:**

Refinement on *F*^2^	Primary atom site location: structure-invariant direct methods
Least-squares matrix: full	Secondary atom site location: difference Fourier map
*R*[*F*^2^ > 2σ(*F*^2^)] = 0.023	Hydrogen site location: inferred from neighbouring sites
*wR*(*F*^2^) = 0.062	H-atom parameters constrained
*S* = 1.05	*w* = 1/[σ^2^(*F*_o_^2^) + (0.0323*P*)^2^ + 0.8768*P*] where *P* = (*F*_o_^2^ + 2*F*_c_^2^)/3
4265 reflections	(Δ/σ)_max_ = 0.001
207 parameters	Δρ_max_ = 0.77 e Å^−^^3^
0 restraints	Δρ_min_ = −1.12 e Å^−^^3^

### Special details

**Table:**

Geometry. All e.s.d.'s (except the e.s.d. in the dihedral angle between two l.s. planes) are estimated using the full covariance matrix. The cell e.s.d.'s are taken into account individually in the estimation of e.s.d.'s in distances, angles and torsion angles; correlations between e.s.d.'s in cell parameters are only used when they are defined by crystal symmetry. An approximate (isotropic) treatment of cell e.s.d.'s is used for estimating e.s.d.'s involving l.s. planes.
Refinement. Refinement of *F*^2^ against ALL reflections. The weighted *R*-factor *wR* and goodness of fit *S* are based on *F*^2^, conventional *R*-factors *R* are based on *F*, with *F* set to zero for negative *F*^2^. The threshold expression of *F*^2^ > σ(*F*^2^) is used only for calculating *R*-factors(gt) *etc*. and is not relevant to the choice of reflections for refinement. *R*-factors based on *F*^2^ are statistically about twice as large as those based on *F*, and *R*- factors based on ALL data will be even larger.

### Fractional atomic coordinates and isotropic or equivalent isotropic displacement parameters (Å^2^)

**Table:**

	*x*	*y*	*z*	*U*_iso_*/*U*_eq_	Occ. (<1)
Sn1	0.5000	0.5000	0.5000	0.01198 (6)	
Br1	0.43772 (3)	0.21868 (2)	0.45713 (2)	0.01709 (7)	
Br2	0.75993 (3)	0.43138 (3)	0.38027 (2)	0.01756 (7)	
Br3	0.77314 (5)	0.20503 (4)	0.97712 (4)	0.02952 (10)	0.50
Cl2	0.77314 (5)	0.20503 (4)	0.97712 (4)	0.02952 (10)	0.50
Cl1	0.13889 (10)	0.39025 (9)	−0.06015 (7)	0.03579 (19)	
N1	−0.1837 (3)	0.2294 (2)	0.5641 (2)	0.0186 (4)	
H1	−0.2525	0.2675	0.5294	0.022*	
N2	0.1383 (3)	0.0420 (2)	0.7154 (2)	0.0210 (5)	
C1	0.3775 (3)	0.4724 (2)	0.3230 (2)	0.0151 (5)	
C2	0.4409 (3)	0.5295 (3)	0.2416 (2)	0.0184 (5)	
H2	0.5368	0.5838	0.2658	0.022*	
C3	0.3651 (3)	0.5080 (3)	0.1248 (3)	0.0229 (6)	
H3	0.4078	0.5482	0.0694	0.027*	
C4	0.2268 (3)	0.4272 (3)	0.0906 (2)	0.0240 (6)	
C5	0.1589 (3)	0.3723 (3)	0.1713 (3)	0.0242 (6)	
H5	0.0623	0.3193	0.1472	0.029*	
C6	0.2349 (3)	0.3965 (3)	0.2886 (2)	0.0194 (5)	
H6	0.1891	0.3609	0.3457	0.023*	
C7	−0.2277 (3)	0.1231 (3)	0.6020 (3)	0.0202 (5)	
H7	−0.3331	0.0915	0.5928	0.024*	
C8	−0.1239 (3)	0.0602 (3)	0.6534 (2)	0.0188 (5)	
H8	−0.1573	−0.0146	0.6797	0.023*	
C9	0.0341 (3)	0.1054 (3)	0.6681 (2)	0.0159 (5)	
C10	0.0741 (3)	0.2198 (3)	0.6290 (2)	0.0173 (5)	
H10	0.1781	0.2558	0.6383	0.021*	
C11	−0.0357 (3)	0.2780 (3)	0.5785 (2)	0.0182 (5)	
H11	−0.0073	0.3545	0.5528	0.022*	
C12	0.3025 (3)	0.0848 (3)	0.7299 (3)	0.0281 (6)	
H12A	0.3274	0.1224	0.6648	0.042*	
H12B	0.3610	0.0023	0.7212	0.042*	
H12C	0.3289	0.1583	0.8134	0.042*	
C13	0.0909 (4)	−0.0632 (3)	0.7687 (3)	0.0308 (7)	
H13A	0.0393	−0.0169	0.8453	0.046*	
H13B	0.1812	−0.1062	0.7896	0.046*	
H13C	0.0204	−0.1371	0.7070	0.046*	
C14	0.5185 (4)	0.1403 (3)	1.0834 (3)	0.0282 (6)	
H14	0.5317	0.2364	1.1396	0.034*	
C15	0.6185 (4)	0.0876 (3)	0.9901 (3)	0.0249 (6)	
C16	0.5999 (4)	−0.0512 (3)	0.9068 (3)	0.0276 (6)	
H16	0.6685	−0.0853	0.8429	0.033*	

### Atomic displacement parameters (Å^2^)

**Table:**

	*U*^11^	*U*^22^	*U*^33^	*U*^12^	*U*^13^	*U*^23^
Sn1	0.01253 (12)	0.01045 (11)	0.01205 (11)	0.00037 (8)	−0.00207 (8)	0.00356 (8)
Br1	0.01696 (13)	0.01231 (11)	0.02127 (13)	−0.00079 (9)	−0.00159 (9)	0.00599 (9)
Br2	0.01604 (13)	0.01762 (12)	0.01965 (13)	0.00199 (9)	0.00089 (9)	0.00756 (10)
Br3	0.0333 (2)	0.0299 (2)	0.0258 (2)	−0.00401 (17)	−0.00423 (17)	0.01252 (17)
Cl2	0.0333 (2)	0.0299 (2)	0.0258 (2)	−0.00401 (17)	−0.00423 (17)	0.01252 (17)
Cl1	0.0435 (5)	0.0421 (4)	0.0144 (3)	0.0164 (3)	−0.0097 (3)	0.0016 (3)
N1	0.0168 (11)	0.0179 (10)	0.0218 (11)	0.0034 (8)	−0.0034 (9)	0.0087 (9)
N2	0.0219 (12)	0.0216 (11)	0.0209 (11)	0.0057 (9)	−0.0017 (9)	0.0094 (9)
C1	0.0183 (12)	0.0117 (10)	0.0125 (11)	0.0033 (9)	−0.0015 (9)	0.0012 (9)
C2	0.0183 (12)	0.0189 (12)	0.0178 (12)	0.0046 (10)	0.0002 (10)	0.0063 (10)
C3	0.0261 (14)	0.0282 (14)	0.0164 (12)	0.0111 (11)	0.0038 (11)	0.0092 (11)
C4	0.0275 (15)	0.0252 (13)	0.0137 (12)	0.0118 (11)	−0.0050 (10)	0.0003 (10)
C5	0.0222 (14)	0.0218 (13)	0.0231 (14)	0.0012 (11)	−0.0085 (11)	0.0032 (11)
C6	0.0188 (13)	0.0193 (12)	0.0179 (12)	−0.0006 (10)	−0.0035 (10)	0.0052 (10)
C7	0.0176 (13)	0.0194 (12)	0.0216 (13)	−0.0016 (10)	−0.0003 (10)	0.0060 (10)
C8	0.0229 (14)	0.0158 (11)	0.0178 (12)	−0.0011 (10)	0.0010 (10)	0.0066 (10)
C9	0.0206 (13)	0.0141 (11)	0.0114 (11)	0.0054 (9)	0.0000 (9)	0.0025 (9)
C10	0.0165 (12)	0.0172 (11)	0.0187 (12)	−0.0010 (9)	0.0004 (10)	0.0075 (10)
C11	0.0209 (13)	0.0161 (11)	0.0181 (12)	−0.0012 (10)	−0.0009 (10)	0.0075 (10)
C12	0.0196 (14)	0.0363 (16)	0.0294 (15)	0.0120 (12)	−0.0009 (12)	0.0124 (13)
C13	0.0401 (18)	0.0283 (15)	0.0313 (16)	0.0090 (13)	−0.0012 (13)	0.0192 (13)
C14	0.0389 (17)	0.0190 (12)	0.0208 (14)	0.0096 (12)	−0.0056 (12)	0.0004 (10)
C15	0.0312 (15)	0.0216 (13)	0.0192 (13)	0.0061 (11)	−0.0089 (11)	0.0055 (11)
C16	0.0338 (16)	0.0253 (14)	0.0197 (13)	0.0110 (12)	−0.0019 (12)	0.0028 (11)

### Geometric parameters (Å, °)

**Table:**

Sn1—C1^i^	2.148 (3)	C5—H5	0.9500
Sn1—C1	2.148 (3)	C6—H6	0.9500
Sn1—Br2^i^	2.7172 (9)	C7—C8	1.357 (4)
Sn1—Br2	2.7172 (8)	C7—H7	0.9500
Sn1—Br1	2.7319 (7)	C8—C9	1.418 (4)
Sn1—Br1^i^	2.7319 (7)	C8—H8	0.9500
Br3—C15	1.807 (3)	C9—C10	1.420 (3)
Cl1—C4	1.744 (3)	C10—C11	1.357 (4)
N1—C11	1.344 (3)	C10—H10	0.9500
N1—C7	1.346 (3)	C11—H11	0.9500
N1—H1	0.8800	C12—H12A	0.9800
N2—C9	1.337 (3)	C12—H12B	0.9800
N2—C13	1.460 (4)	C12—H12C	0.9800
N2—C12	1.465 (4)	C13—H13A	0.9800
C1—C2	1.386 (4)	C13—H13B	0.9800
C1—C6	1.392 (4)	C13—H13C	0.9800
C2—C3	1.392 (4)	C14—C16^ii^	1.378 (5)
C2—H2	0.9500	C14—C15	1.389 (4)
C3—C4	1.382 (4)	C14—H14	0.9500
C3—H3	0.9500	C15—C16	1.379 (4)
C4—C5	1.383 (4)	C16—C14^ii^	1.378 (5)
C5—C6	1.391 (4)	C16—H16	0.9500
			
C1^i^—Sn1—C1	180.0	C1—C6—H6	119.7
C1^i^—Sn1—Br2^i^	89.62 (7)	N1—C7—C8	121.0 (2)
C1—Sn1—Br2^i^	90.38 (7)	N1—C7—H7	119.5
C1^i^—Sn1—Br2	90.38 (7)	C8—C7—H7	119.5
C1—Sn1—Br2	89.62 (7)	C7—C8—C9	120.5 (2)
Br2^i^—Sn1—Br2	180.0	C7—C8—H8	119.8
C1^i^—Sn1—Br1	89.88 (7)	C9—C8—H8	119.8
C1—Sn1—Br1	90.12 (7)	N2—C9—C8	121.3 (2)
Br2^i^—Sn1—Br1	91.55 (3)	N2—C9—C10	122.5 (2)
Br2—Sn1—Br1	88.45 (3)	C8—C9—C10	116.2 (2)
C1^i^—Sn1—Br1^i^	90.12 (7)	C11—C10—C9	120.2 (2)
C1—Sn1—Br1^i^	89.88 (7)	C11—C10—H10	119.9
Br2^i^—Sn1—Br1^i^	88.45 (3)	C9—C10—H10	119.9
Br2—Sn1—Br1^i^	91.55 (3)	N1—C11—C10	121.3 (2)
Br1—Sn1—Br1^i^	180.0	N1—C11—H11	119.4
C11—N1—C7	120.7 (2)	C10—C11—H11	119.4
C11—N1—H1	119.6	N2—C12—H12A	109.5
C7—N1—H1	119.6	N2—C12—H12B	109.5
C9—N2—C13	120.7 (2)	H12A—C12—H12B	109.5
C9—N2—C12	122.5 (2)	N2—C12—H12C	109.5
C13—N2—C12	116.4 (2)	H12A—C12—H12C	109.5
C2—C1—C6	119.5 (2)	H12B—C12—H12C	109.5
C2—C1—Sn1	120.03 (18)	N2—C13—H13A	109.5
C6—C1—Sn1	120.51 (19)	N2—C13—H13B	109.5
C1—C2—C3	120.5 (3)	H13A—C13—H13B	109.5
C1—C2—H2	119.7	N2—C13—H13C	109.5
C3—C2—H2	119.7	H13A—C13—H13C	109.5
C4—C3—C2	118.9 (3)	H13B—C13—H13C	109.5
C4—C3—H3	120.6	C16^ii^—C14—C15	119.1 (3)
C2—C3—H3	120.6	C16^ii^—C14—H14	120.4
C3—C4—C5	121.8 (3)	C15—C14—H14	120.4
C3—C4—Cl1	118.7 (2)	C16—C15—C14	121.1 (3)
C5—C4—Cl1	119.5 (2)	C16—C15—Br3	119.9 (2)
C4—C5—C6	118.6 (3)	C14—C15—Br3	119.0 (2)
C4—C5—H5	120.7	C14^ii^—C16—C15	119.7 (3)
C6—C5—H5	120.7	C14^ii^—C16—H16	120.1
C5—C6—C1	120.6 (3)	C15—C16—H16	120.1
C5—C6—H6	119.7		
			
C1^i^—Sn1—C1—C2	19 (100)	C2—C1—C6—C5	2.8 (4)
Br2^i^—Sn1—C1—C2	134.73 (19)	Sn1—C1—C6—C5	−176.7 (2)
Br2—Sn1—C1—C2	−45.27 (19)	C11—N1—C7—C8	−1.4 (4)
Br1—Sn1—C1—C2	−133.72 (19)	N1—C7—C8—C9	0.0 (4)
Br1^i^—Sn1—C1—C2	46.28 (19)	C13—N2—C9—C8	−8.1 (4)
C1^i^—Sn1—C1—C6	−161 (100)	C12—N2—C9—C8	179.0 (2)
Br2^i^—Sn1—C1—C6	−45.7 (2)	C13—N2—C9—C10	172.4 (2)
Br2—Sn1—C1—C6	134.3 (2)	C12—N2—C9—C10	−0.5 (4)
Br1—Sn1—C1—C6	45.8 (2)	C7—C8—C9—N2	−178.2 (2)
Br1^i^—Sn1—C1—C6	−134.2 (2)	C7—C8—C9—C10	1.3 (4)
C6—C1—C2—C3	−1.9 (4)	N2—C9—C10—C11	178.2 (2)
Sn1—C1—C2—C3	177.68 (19)	C8—C9—C10—C11	−1.3 (4)
C1—C2—C3—C4	−0.9 (4)	C7—N1—C11—C10	1.4 (4)
C2—C3—C4—C5	2.8 (4)	C9—C10—C11—N1	0.0 (4)
C2—C3—C4—Cl1	−175.6 (2)	C16^ii^—C14—C15—C16	0.7 (5)
C3—C4—C5—C6	−1.8 (4)	C16^ii^—C14—C15—Br3	179.7 (2)
Cl1—C4—C5—C6	176.5 (2)	C14—C15—C16—C14^ii^	−0.7 (5)
C4—C5—C6—C1	−1.0 (4)	Br3—C15—C16—C14^ii^	−179.7 (2)

Symmetry codes: (i) −*x*+1, −*y*+1, −*z*+1; (ii) −*x*+1, −*y*, −*z*+2.
